# The Role of Embryonic Chick Muscle Cell Culture in the Study of Skeletal Myogenesis

**DOI:** 10.3389/fphys.2021.668600

**Published:** 2021-05-20

**Authors:** Manoel L. Costa, Arnon D. Jurberg, Claudia Mermelstein

**Affiliations:** ^1^Laboratório de Diferenciação Muscular, Instituto de Ciências Biomédicas, Universidade Federal do Rio de Janeiro, Rio de Janeiro, Brazil; ^2^Faculdade de Medicina-Presidente Vargas, Universidade Estácio de Sá, Rio de Janeiro, Brazil

**Keywords:** myogenesis, chick embryo, skeletal muscle, muscle differentiation, myoblast, myotube

## Abstract

The mechanisms involved in the development of skeletal muscle fibers have been studied in the last 70 years and yet many aspects of this process are still not completely understood. A myriad of *in vivo* and *in vitro* invertebrate and vertebrate animal models has been used for dissecting the molecular and cellular events involved in muscle formation. Among the most used animal models for the study of myogenesis are the rodents rat and mouse, the fruit fly Drosophila, and the birds chicken and quail. Here, we describe the robustness and advantages of the chick primary muscle culture model for the study of skeletal myogenesis. In the myoblast culture obtained from embryonic chick pectoralis muscle it is possible to analyze all the steps involved in skeletal myogenesis, such as myoblast proliferation, withdrawal from cell cycle, cell elongation and migration, myoblast alignment and fusion, the assembly of striated myofibrils, and the formation of multinucleated myotubes. The fact that *in vitro* chick myotubes can harbor hundreds of nuclei, whereas myotubes from cell lines have only a dozen nuclei demonstrates the high level of differentiation of the autonomous chick myogenic program. This striking differentiation is independent of serum withdrawal, which points to the power of the model. We also review the major pro-myogenic and anti-myogenic molecules and signaling pathways involved in chick myogenesis, in addition to providing a detailed protocol for the preparation of embryonic chick myogenic cultures. Moreover, we performed a bibliometric analysis of the articles that used this model to evaluate which were the main explored topics of interest and their contributors. We expect that by describing the major findings, and their advantages, of the studies using the embryonic chick myogenic model we will foster new studies on the molecular and cellular process involved in muscle proliferation and differentiation that are more similar to the actual *in vivo* condition than the muscle cell lines.

## A Brief View of Skeletal Myogenesis

**FIGURE 1:**
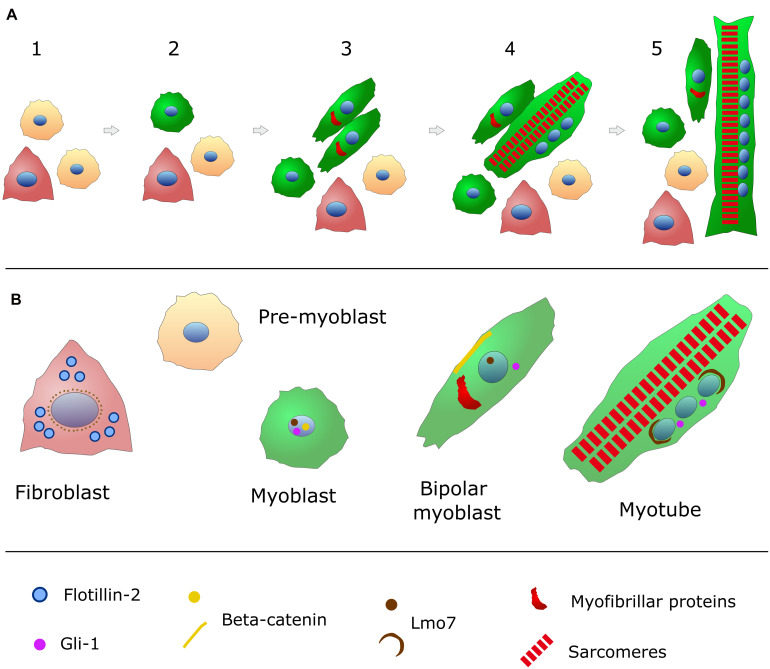
Schematic representation of *in vitro* chick embryonic skeletal myogenesis. **(A)** Specific cell phenotypes can be characterized during skeletal muscle development (for more details, see Table 1). Presumptive myoblasts (or pre-myoblasts, shown in beige) are mononucleated (nuclei in blue), round shaped and proliferative cells that do not express myofibrillar proteins. When these cells withdraw cell cycle, they begin to express desmin (green in the cytoplasm) and they change to a bipolar shape. These bipolar myoblasts begin to express myofibrillar proteins (red) and they will align with other myoblasts. Cell adhesion and fusion happens between steps 3 and 5. Muscle differentiation culminates with the formation of long and multinucleated myotubes filled (steps 4 and 5) with contractile myofibrils (sarcomeres in red). Pre-myoblasts (beige cells), post-mitotic myoblasts (round green cells), bipolar myoblasts (elongated green cells), and fibroblasts (triangular-shaped and carmine-colored cells) are present in all steps of chick myogenic cultures (steps 1–5). **(B)** The intracellular distribution of the structural and signaling proteins flotillin-2 (light blue), beta-catenin (yellow), Lmo7 (brown), and myofibrillar proteins (red) are shown for each specific cell phenotype (myoblasts, fibroblasts and myotubes) found in chick myogenic cell cultures.

**FIGURE 2:**
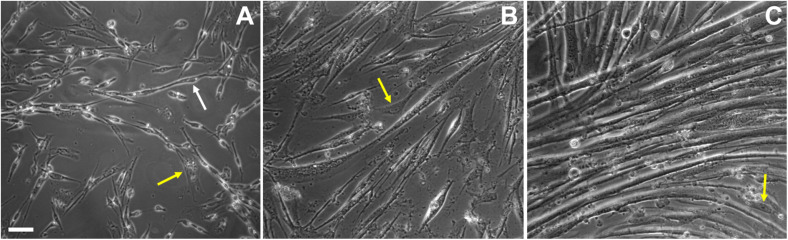
Cell shape changes during *in vitro* chick myogenesis. Primary cultures of 11-day old chick myoblasts were grown for 24, 48, and 72 h and images were acquired under phase contrast microscopy. Bipolar myoblasts aligned in a pre-fusion chain-like structure (white arrow) are seen in a 24-h culture **(A)**, while thin myotubes are already present in a 48-h culture **(B)** and multinucleated myotubes are present in a 72-h culture **(C)**. Note the presence of fibroblasts in all the images (**A–C**, yellow arrows). Scale bar = 10 μm.

**FIGURE 3:**
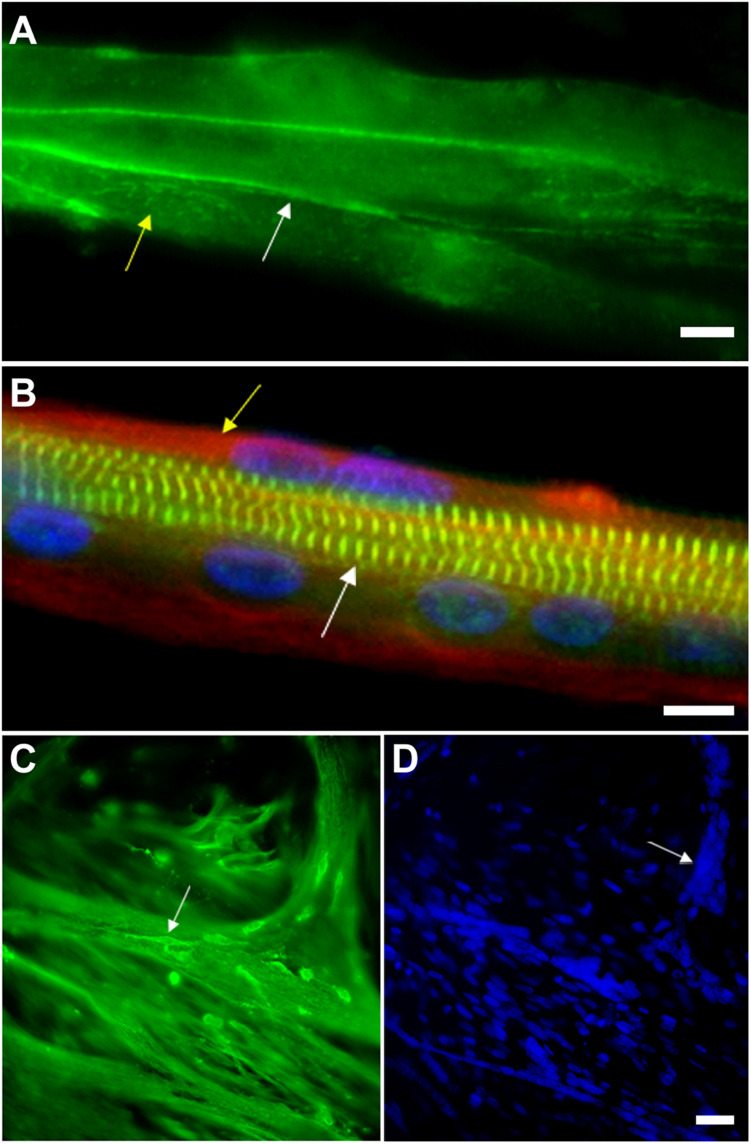
Myoblast adhesion and myotube formation are hallmarks of skeletal myogenesis. **(A)** Cadherin and beta-catenin accumulate at cell-cell adhesion contacts in pre-fusion chick myoblasts. In chick myogenic cultures 24 h after cell plating, primary fusion (myoblast-myoblast fusion) can be observed as an intense immunolabeling of beta-catenin (green, white arrow in **A**) in continuous lines at cell-cell contacts. Note that beta-catenin is also present in a dot-like pattern in the whole sarcolemma (green, yellow arrow in **A**). Scale bar in **(A)** = 10 μm. **(B)** Myotubes from chick primary skeletal muscle cultures are multinucleated cells that contain highly organized myofibrils. Chick skeletal muscle cells were grown in culture for 72 h and stained with antibodies anti-sarcomeric alpha-actinin (green), anti-desmin (red, yellow arrow in **B**) and with the nuclear dye DAPI (blue). Note in the merged image the periodical labeling of alpha-actinin in the Z-bands of myofibrils (white arrow in **B**), the presence of desmin filaments at the periphery of the myotube (yellow arrow in **B**) and well-aligned nuclei. Scale bar in *B* = 10 μm. **(C,D)** Myoblasts adhere to myotubes in a secondary fusion process. Chick skeletal muscle cells were grown in culture for 72 h and stained with antibodies anti-alpha-tubulin (green, **C**) and with the nuclear dye DAPI (blue, **D**). Small mononucleated myoblasts (white arrow in **C**) can be seen at the top of a multinucleated myotube, which is evidenced by the microtubule staining. DAPI shows the presence of a high number of nuclei in each chick myotube cell (blue, **D**). Image **(A)** shows a 24-h primary myogenic culture, where most of the myoblasts are undergoing primary myoblast fusion (myoblast-myoblast fusion), while image **(C)** shows a 72-h primary myogenic culture, where secondary myoblast fusion (myoblast-myotube fusion) is frequently observed. Scale bar in **(D)** = 20 μm.

Among the various processes involved in the organogenesis of vertebrates, the formation of skeletal muscle (i.e., skeletal myogenesis) initiates in the paraxial mesoderm of the developing embryo, which gradually segments into somites (Christ and Ordahl, 1995; Chal and Pourquié, 2017). Epithelial-derived mesenchymal cells give rise to round proliferative presumptive mononucleated myoblasts. Signaling molecules, mainly from the Wnt/beta-catenin, Sonic hedgehog (Shh), Bone morphogenetic protein (BMP), and Notch signaling pathways, participate in the initial steps of embryonic myogenesis (Cossu et al., 1996; Ikeya and Takada, 1998; Goichberg et al., 2001; Elia et al., 2007; Wang et al., 2010; Bentzinger et al., 2012; Maltzahn et al., 2012). Presumptive mononucleated myoblasts, which do not express myofibrillar proteins, will proliferate a few rounds of replication, and exit cell cycle. The activation of the muscle specific-master switch genes MyoD and Myf5 leads to the differentiation of presumptive myoblasts into elongated post-mitotic myoblasts (Davis et al., 1987; Choi et al., 1990). MyoD and Myf5, in conjunction with Myogenin and MRF4, are highly conserved genes known as myogenic regulatory factors (MRFs). They are organized into hierarchical gene expression networks that control skeletal muscle differentiation (Bentzinger et al., 2012). For instance, their activation drives the expression of muscle specific and myofibrillar proteins (Holtzer et al., 1991; Costa et al., 2004). Elongated post-mitotic myoblasts (or bipolar myoblasts) adhere to each other through a cell membrane-dependent recognition event, which is subsequently followed by cell fusion (that is, “primary fusion”). Myoblast fusion relies on membrane proteins, mainly cadherins, myomaker, and myomerger, to form multinucleated myotubes (Zeschnigk et al., 1995; Costa, 2014; Sampath et al., 2018; Figures 1–3 and Supplementary Figure 1). Initially, the nuclei of myotubes are centrally placed, but they migrate to the periphery (Fear, 1977) while striated actomyosin-based myofibrils organize within the sarcoplasm of the multinucleated syncytium (Figures 1–3 and Table 1). The growth of myotubes results from a secondary phase of cell fusion, which involves the fusion of mononucleated myoblasts into the multinucleated myotubes. During the formation of mature multinucleated myotubes, myofibrillar proteins start to organize into long contractile structures encompassing several muscle-specific proteins, such as actin, myosin, troponins C/T/I, tropomyosin, alpha-actinin, titin, nebulin, desmin, myosin binding protein-C (MyBP-C), myomesin, and tropomodulin (Figures 1–3 and Table 1).

**TABLE 1:** Characteristics of specific cell phenotypes during chick skeletal myogenesis.

	Presumptive myoblast	Round myoblast	Bipolar myoblast	Striated myoblast	Myotube
Proliferation status	Proliferative	Non-proliferative	Non-proliferative	Non-proliferative	Non-proliferative

Desmin expression	Desmin-negative	Desmin-positive	Desmin-positive	Desmin-positive	Desmin-positive

Myofibrillar proteins expression	Myofibrillar proteins-negative	Myofibrillar proteins-negative	Myofibrillar proteins-positive	Myofibrillar proteins-positive	Myofibrillar proteins-positive

Number of nuclei	mononucleated	mononucleated	mononucleated	Mononucleated	multinucleated

Presence of striations	Non-striated	Non-striated	Non-striated	Striated	Striated

Cell shape	Round shaped	Round shaped	Bipolar	Bipolar	Elongated

*The specific cell phenotypes that appear during an in vitro primary culture of embryonic chick skeletal muscle cells are characterized by specific events shown in this Table (for more details, see Figure 1). The differences between cell phenotypes depicted here are the replication capacity, expression of the muscle-specific intermediate filament desmin, expression of myofibrillar proteins, the number of nuclei per cell, presence of striations, and cell shape.*

Different animal models have been used for dissecting the above-described steps of skeletal muscle differentiation, including rodents (mouse and rat), fishes, fruit-flies, frogs, and birds (chicken and quail), as well as human muscle cells. Among these models, the chick embryo is one of the major contributors of our current knowledge of muscle development (Buckingham et al., 2003). The chick embryonic skeletal muscle primary culture is a robust *in vitro* model for the study of skeletal muscle differentiation because of its autonomous myogenic program (Holtzer et al., 1991; Mermelstein et al., 1996; Oliveira et al., 2015; Ojima et al., 2016; Rosa de Andrade et al., 2018). All the before mentioned steps of skeletal myogenesis can be observed in the chick muscle cultures. Skeletal myogenesis proceeds in chick myoblast cultures through the following main sequential and temporal stages (some of which can be seen in Figures 1–3 and Table 1): myoblast proliferation, cell cycle withdrawal, myoblast elongation and migration, myoblast alignment and fusion, and the formation of multinucleated myotubes, as well as the formation of contractile myofibrils.

Studies using chick myogenic cultures led to the discovery of many soluble factors that either stimulate or inhibit myoblast fusion and/or myotube formation, and which can be categorized into pro- or anti-myogenic. Table 2 shows activators and inhibitors of embryonic chick skeletal myogenesis. Interestingly, there is a greater number of known molecules (∼3 fold) that inhibit myogenesis as compared to the ones that produced stimulatory outcomes. This difference can be explained by (i) the incompleteness of available knowledge, since it is usually easier to identify an inhibition or blockage of myofiber formation than a positive, sometimes subtle effect, and/or (ii) researchers in the field may be more interested in inhibitory molecules rather than stimulatory ones. Together, the information collected in Table 2 could be useful to build a more complete picture of the molecular machinery controlling skeletal myogenesis.

**TABLE 2:** Pro- or anti-myogenic effects of different molecules/substances during *in vitro* chick skeletal myogenesis.

Substance name	Substance activity	Substance concentration	References
**Enhance myoblast fusion and/or muscle differentiation**			
Alpha-cyclodextrin	Membrane phospholipid depleting agent	2 mM	Possidonio et al., 2011
6-Bromoindirubin-30-oxime (BIO)	GSK3b inhibitor	5 μM	Portilho et al., 2012
Interleukine 4 (IL-4)	Binds to IL-4 receptor	10 ng/mL	Possidonio et al., 2011
Isoproterenol (ISO)	Beta-adrenergic receptor agonist	100 nM	Bastos et al., 2019
Methyl-beta-cyclodextrin (MbCD)	Membrane cholesterol depleting agent	2 mM	Mermelstein et al., 2005b, 2007; Portilho et al., 2007, 2012
Wnt-3a	Canonical Wnt/beta-catenin pathway activator	L-Wnt3a-cells conditioned media	Portilho et al., 2007
**Inhibits myoblast fusion and/or muscle differentiation**			
Chloroquine Lys05	Lysosomal function inhibitor	1 μM	Bagri et al., 2020
4-{[2-[(2-Cyanobenzyl) thio]-4-oxothieno[3,2-d]pyrimidin-3(4H)-yl]methyl}benzoic acid (C3)	S-nitrosoglutathione reductase (GSNOR) inhibitor	10 μM	Yamashita et al., 2017
Cytochalasin B	Actin polymerization inhibitor	5 μM	Ojima et al., 2016
Dickkopf-related protein 1 (Dkk-1)	canonical Wnt/beta-catenin inhibitor	0.1 mg/mL	Portilho et al., 2012
Ethylene glycol tetraacetic acid (EGTA)	Calcium chelator	1.8 mM	Paterson and Strohman, 1972; Mermelstein et al., 2005a
Forskolin	Adenylate cyclase activator	100 μM	Baek et al., 1994
Ouabain	Na^+^/K^+^-ATPase inhibitor	10 μM	Oliveira et al., 2015
PD150606	Calpain inhibitor	20 μM	Buffolo et al., 2015
Rapamycin	mTOR signaling pathway inhibitor	3 μM	Bastos et al., 2019
Simvastatin	Cholesterol biosynthetic pathway inhibitor	0.5 μM	Portilho et al., 2012
Soluble frizzled receptor (Frzb-1)	Wnt pathway inhibitor	L-Fzrb1-cells conditioned media	Mermelstein et al., 2007
Sonic hedhehog (Shh)	Shh pathway activator	10 ng	Teixeira et al., 2018
12-O-Tetradecanoylphorbol-13-acetate (TPA)	protein kinase C activator	10^–7^ M	Cohen et al., 1977
Trifluoperazine (TFP)	Calmodulin antagonist	10 μM	Bar-Sagi and Prives, 1983
U0126	MEK-ERK pathway inhibitor	10 μM	Oliveira et al., 2015
Wnt-5a	Non-canonical Wnt pathway activator	L-Wnt5a-cells conditioned media	Portilho et al., 2007; Bastos et al., 2019

*Only studies using embryonic chick muscle cultures obtained from pectoral (breast) skeletal muscle were included in this table. Studies using embryonic chick leg muscles were not included. Nearly all the studies described in the table used the indicated concentrations of substances over a 24-h period of incubation in vitro.*

The use of chick cells as a model of *in vitro* myogenesis has largely contributed for the description and molecular characterization of the aforementioned steps during myotube formation. In the section below, we will detail its main advantages, disadvantages, and particularities as an experimental model for skeletal myogenesis.

## Dissecting Skeletal Myogenesis Using Chick Myoblast Cultures

The characterization and quantification of different aspects of skeletal myogenesis is relatively simple to achieve in culture of chick myoblasts (Abrunhosa et al., 2014; Bastos et al., 2019; Bagri et al., 2020). Among them, information regarding the number of mononucleated myoblasts and multinucleated myotubes, as well as fibroblasts, the number of nuclei within each myotube, area of muscle cells, and size of myotubes can be acquired from image processing tools, such as ImageJ (Schindelin et al., 2012). From these data, it is also possible to compute both the fusion index, which refers to the number of nuclei in myotubes divided by the total number of nuclei in a microscopy field of view, and the differentiation index, which is obtained from the number of nuclei in myosin heavy chain (MyHC)-positive cells divided by the total number of nuclei in a field. In this regard, the staining of MyHC can be replaced by other myofibrillar protein, such as desmin, alpha-actinin, troponin, tropomyosin, or titin.

One important aspect of chick skeletal myoblasts grown *in vitro* is their autonomous muscle differentiation program. Whereas the exposition of the mouse BC3H1 cell line to mitogens caused the downregulation of sarcomeric protein synthesis and cell cycle reentry (Taubman et al., 1989), chick muscle cells appear not to be subject to the exact same molecular mechanisms. This is reflected in the non-reliance of serum starvation to trigger chick myoblast differentiation into myotubes, unlike immortalized mice, rats, and human cells (Lawson and Purslow, 2000). For this reason, assays using chick myoblasts are the most suitable for tracking morphological and protein/gene expression changes over time during skeletal myogenesis. An example of this kind of studies is the analysis of the sequence of expression of muscle-specific proteins in muscle cells during differentiation in culture (Lin et al., 1994). Upon terminal cell division, chick myoblasts start to express genes of myofibrillar and structural proteins in a specific order before cell fusion. Desmin is the first structural muscle-specific protein to appear after cell cycle withdrawal and it is followed by alpha-actin, troponin-I, alpha-actinin, MyHC, titin, and nebulin in an apparently stochastic order (Lin et al., 1994). In the period between 15 and 24 h after cell cycle exit, these contractile proteins first organize into nonstriated myofibrils and them into striated myofibrils, concurrently with the expression of myomesin and MyBP-C in the developing A-bands (Lin et al., 1994).

One of the most studied and yet not completely understood step of skeletal muscle differentiation is myoblast fusion, which is a complex process regulated by several molecules and pathways (Figures 1–3 and Supplementary Figure 1). Myoblast fusion in chick cells occurs after 50–65 h of culture, but it takes about 6 h after the first cell-to-cell contact begin. Cell fusion usually follows an end-to-end rather than side-to-side alignment of myoblasts (Fear, 1977). Lipids, proteins, ions, and cell organelles have been implicated in the fusion of chick myoblasts. Together, they are part of an extensive and growing list, which includes cholesterol, sphingosine, inositol phospholipid, cadherins, calpains, monensin, myomaker, protein kinase C, acetylcholine receptors, ascorbic acid, okadaic acid, nitric oxide synthase, Na^+^-K^+^-ATPase, prostaglandin, concanavalin A, small GTPases, cyclic-AMP, cyclic-GMP, interleukin 4 (IL-4), fibroblast growth factors (FGF), phorbol 12-myristate 13-acetate (TPA), hyaluronic acid, transglutaminase, calcium, zinc, cesium, oxygen, vesicles, and the Golgi apparatus (Shimada, 1971; Knudsen and Horwitz, 1977; Neff et al., 1984; Mermelstein et al., 2005b; Possidonio et al., 2011; Buffolo et al., 2015; Sieiro et al., 2017). The above list is extensive, at least in part, because it contains isolated members (membranar, cytoplasmic, effector and/or nuclear molecules) of the same intracellular signaling pathways.

Among all these aforementioned players, the membrane lipid cholesterol is perhaps the one we know best about its role in myoblast fusion (van der Bosch et al., 1973; Cornell et al., 1980; Sekiya et al., 1984; Hirayama et al., 2001; Nakanishi et al., 2001). Cholesterol is a multifunctional molecule involved in plasma membrane fluidity and permeability, in addition to take part of the synthesis of steroid hormones and others biological processes. It also plays a role in the formation and maintenance of membrane microdomains, which act as signaling centers (Simons and Sampaio, 2011). Our group have studied the role of cholesterol and cholesterol-enriched lipid domains in the sarcolemma of chick myoblast cells by using the cholesterol depletion drug methyl-beta-cyclodextrin (MbCD). This drug shows high affinity for membrane cholesterol, being widely used to extract this molecule from the cell plasma membrane (Ilangumaran and Hoessli, 1998). Upon MbCD treatment, the disorganization of lipid rafts induced an increase in myoblast proliferation and fusion, which led to the formation of larger spontaneously contracting multinucleated myotubes (Mermelstein et al., 2005b, 2007; Portilho et al., 2012). Interestingly, the depletion of cholesterol induced the translocation of beta-catenin into the nuclei of myoblasts and the activation of the canonical Wnt/beta-catenin pathway (Mermelstein et al., 2007; Portilho et al., 2007). A transcriptome analysis of MbCD-treated chick muscle cells revealed multiple changes, involving cell proliferation (cell cycle and p53 signaling), cell adhesion and cytoskeleton (focal adhesion, tight junctions, adherens junctions, gap junctions, and actin cytoskeleton), membrane trafficking-related processes (phagosome, lysosome, and endocytosis), and cell death (apoptosis and autophagy). Among all transcripts that were affected by cholesterol depletion, the levels of Lim domain only protein 7 (Lmo7) mRNA were the most upregulated (Possidonio et al., 2014a, 2016). Lmo7 is a multifunctional protein that can be found in the nucleus, cytoplasm and/or at adhesion junctions in many tissues (Figure 1), with high levels of expression in skeletal muscle, where it has a role as a transcription factor regulating the expression of many skeletal muscle genes, including Pax3, Pax7, MyoD, and Myf5 (Holaska et al., 2006; Possidonio et al., 2016).

Recently, two muscle-specific membrane proteins, myomaker and myomixer, have been identified as fusogenic regulators in vertebrates (Millay et al., 2013; Luo et al., 2015; Bi et al., 2017; Chen et al., 2020). Both myomaker and myomixer have the capacity to directly control the myogenic fusion process. While the experimental deletion of myomaker blocks myoblast fusion in cultured muscle cells and in zebrafish, it does not seem to be involved with other myogenic processes. Mutant myomaker gene causes the human myopathy Carey-Fineman-Ziter syndrome (Di Gioia et al., 2017). Myomaker is regulated by MyoD and myogenin, and its inhibition by miR-140-3p blocks chick myoblast fusion (Luo et al., 2015). Myomixer (also called myomerger or minion) is a small transmembrane peptide that when disrupted prevents myoblast fusion in knockout mice but its forced expression in non-muscle cells, together with myomaker, promotes cell fusion (Bi et al., 2017). Myomixer is also regulated by MyoD and myogenin and its expression pattern is similar to myomaker. It is likely that myomaker and myomixer work together, myomaker initiating myoblast fusion and myomixer expanding the membrane pore (Chen et al., 2020).

One aspect of the transition between myoblast fusion and myotube formation that is of particular interest is the movement and positioning of nuclei derived from mononucleated myoblasts into newly formed multinucleated fibers (Elhanany-Tamir et al., 2012; Folker and Baylies, 2013; Volk, 2013; Liu et al., 2020). Interestingly, Elhanany-Tamir et al. (2012) demonstrated a direct link between myonuclei positioning and proper muscle function in Drosophila, which relies on a network of polarized astral microtubules and KASH proteins that enables the dynamic movement and uniform spacing between the nuclei in each muscle fiber. Earlier studies revealed that nuclei of myotubes can undergo two different movements: (i) translocation along the long axis of the cell, and (ii) three-dimensional rotation in either clockwise or counterclockwise directions (Capers, 1960; Cooper and Konigsberg, 1961; Englander and Rubin, 1987). The meaning of these nuclear movements is still elusive during skeletal myogenesis, but the perinuclear region is becoming a focus of studies because of its highly dynamic and specialized concentration of molecules. The juxtanuclear area of muscle cells preferentially harbor specific organelles, such as centrosomes and lysosomes, as well as a number of structural and signaling proteins, such as desmin, Gli-1, and Lmo7 (Figure 1; Mermelstein et al., 2006; Possidonio et al., 2016; Teixeira et al., 2018; Bagri et al., 2020).

Interestingly, at the end of skeletal muscle differentiation in primary chick muscle cell cultures, a single myotube may exhibit hundreds of nuclei within its cytoplasm (Figure 3), whereas myotubes formed in cultures of widely used skeletal muscle cell lines, such as mouse C2C12, rat L6 and L8 cells, and human CL25, are usually composed by only a few (2–20) nuclei. As mentioned above, this is probably related to the autonomous nature of the chick muscle differentiation program. In addition, the formation of branched myotubes is also a common observation in chick muscle cell cultures (Konigsberg, 1963), but they have not been reported *in vivo*. Their branching appears to occur when myotubes are not aligned parallel to each other *in vitro*, which produces a contact between the end of one myotube and the lateral surface of another one (Fear, 1977). Furthermore, *in vitro* myotubes differ from *in vivo* myotubes in that the former are relatively hypernucleated (Okazaki and Holtzer, 1966).

After myoblast fusion, sarcomeres assemble to form contractile myofibrils in multinucleated myotubes, in a process called myofibrillogenesis. The formation of a myofibril occurs by the stepwise addition of several different myofibrillar proteins. This complex and dynamic process is based on the conversion of actin microfilament scaffolds decorated with alpha-actinin and other actin-associated proteins into stress fiber-like structures (also called pre-myofibrils; Almenar-Queralt et al., 1999). While these alpha-actinin deposits coalesce into periodically distributed nascent Z lines, they bind to the Z-line epitope of titin. Concomitant with the progressive alignment of Z-line molecules, thick filaments, formed by the muscle isoform of myosin II, are linked to the nascent myofibrils. Lastly, M-lines are assembled with the addition of myomesin and other proteins, including the M-line part of titin (Sanger et al., 2010; Wang et al., 2020). In summary, the assembly of sarcomeres involves interaction between two organizing centers, (i) one involves the assembly of thick filaments and their associated proteins, including titin, and (ii) the other involves the assembly of thin filaments and their associated proteins, forming the I-Z-I complex (Hill et al., 1986). Myofibril assembly is based on force transduction through the attachment of myofibrils to the sarcolemma and, through cell adhesion complexes, to the extracellular matrix.

A great advantage of the primary culture of chick muscle cells, besides its reliability and independence of differentiation stimuli, is the possibility of observation of molecules (proteins, lipids and RNAs) in cells at single cell- and/or at subcellular-levels. In opposition to biochemical analysis, where a whole cell culture extract (a mixture of different cells) is analyzed, in chick muscle cultures, unlabeled or fluorescently labeled-live cells can be observed at high resolution microscopy and provide valuable information of the behavior and structural characteristics of different myogenic cell phenotypes (proliferative myoblasts, post-mitotic myoblasts, fibroblasts, young myotubes, and mature myotubes). Importantly, chick skeletal muscle cell cultures have some disadvantages, such as the lack of widespread genetic manipulations, which are cutting edge approaches in other animal models of myogenesis, such as the fruit fly Drosophila (Bryantsev et al., 2019). Nevertheless, the genetic approach weakness of the chick muscle culture model has been, in part, surpassed by some recent advances in the transfection of chick cultured cells with siRNA targeted to specific proteins and fluorescent/luminescent protein reporter systems (Mermelstein et al., 2007; Possidonio et al., 2016; Yamashita et al., 2017; Bagri et al., 2020; de Andrade Rosa et al., 2020).

It is important to mention that chick primary muscle cell cultures have been used in the last 70 years as a two-dimensional (2D) layer of cells, and a recent study reported a promising work using three-dimensional (3D) chick skeletal muscle cell cultures to promote muscle differentiation (Gupta et al., 2021). These authors harvested thigh muscle cells from 10-day-old chick embryos, seeded them onto gelatin hydrogels and observed that myoblasts fused into aligned, multinucleated myotubes with robust sarcomere formation in long-term cultures. These results open new venues for engineered *in vitro* models of skeletal muscle which could be useful as platforms for muscle tissue development, function, disease, injury, and drug responses in a controlled setting.

## A Detailed Protocol for Culturing Chick Muscle Cells

**FIGURE 4:**
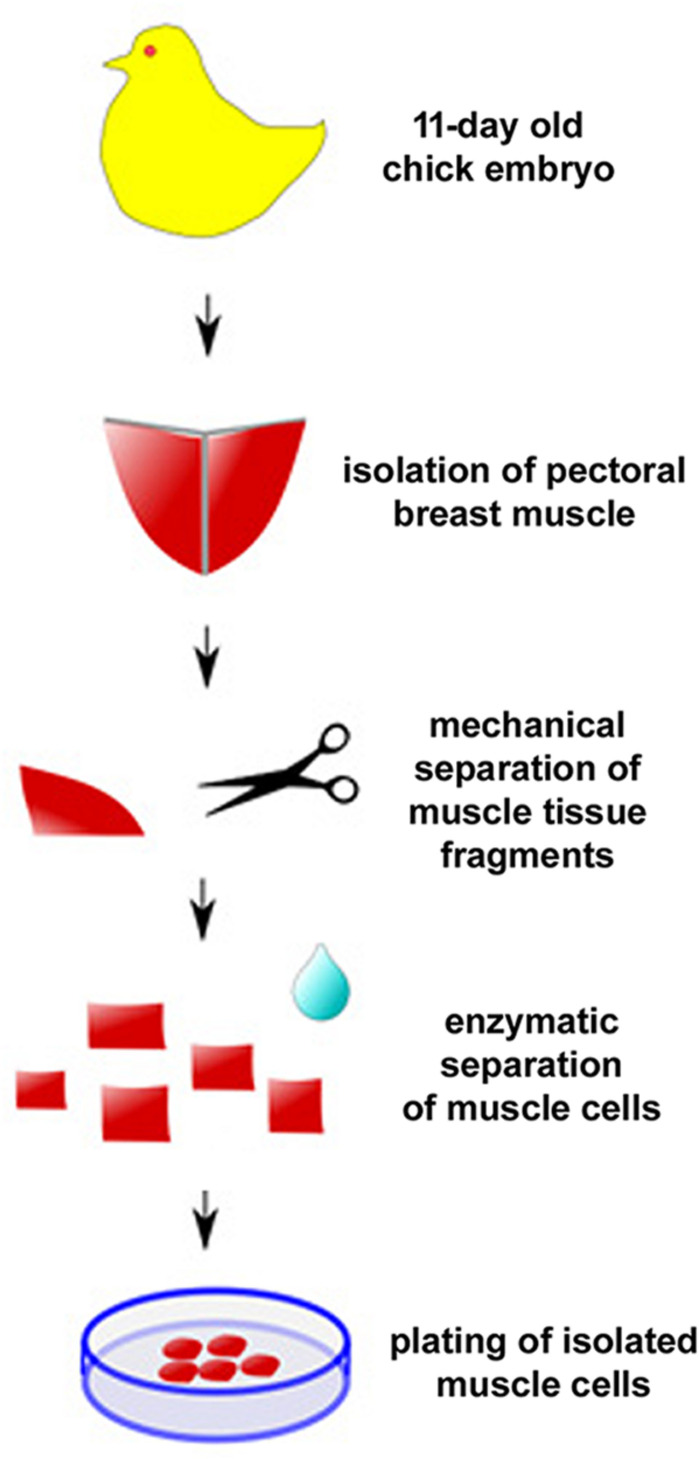
Protocol for primary culture of embryonic chick myogenic cells. Schematic representation of the main steps involved in the culture of embryonic chick pectoral breast muscle cells.

One of the first protocols described for the culture of primary chick embryonic muscle cells is quite simple and therefore widely used (Figure 4; Haba et al., 1966). Briefly, pectoral muscle tissue is removed from 11-day old chicken embryos (usually obtained from a certified poultry farm) with the use of fine watchmaker forceps (N° 5). It is crucial to use all sterile materials and reagents, being extremely careful during the entire cell culture procedure to avoid unwanted contaminants (mostly, bacteria, fungi, or mycoplasma). After separation of the connective tissue under a dissecting scope, muscle tissue is minced with surgery knives and digested with 0.025% trypsin at 37°C for 20–30 min in a 5% CO_2_ incubator. To stop the digestion, a small volume of 8-1-0.5 medium (Minimum Essential Medium with 10% horse serum, 0.5% chick embryo extract, 1% L-glutamine, and 1% penicillin-streptomycin) is added to the sample, which is then centrifuged at 300 × g for 5 min. After removing the supernatant, the pellet is suspended in 8-1-0.5 medium and filtered through a 70 μm nylon cell strainer to produce a mononucleated cell suspension. Isolated mononucleated cells are counted using a hemocytometer chamber and then plated onto collagen-coated dishes, flasks, multi-well, or coverslips for optimal adhesion. In our experience, an initial density of 7.5 × 10^5^ cells/35 mm culture dish in 2 mL of 8-1-0.5 medium is ideal for muscle differentiation. After the first 24 h after plating, cultures are fed daily with fresh 8-1-0.5 medium and kept in a humidified 5% CO_2_ atmosphere at 37°C. It is important to observe muscle cultures every day under a phase contrast microscope to evaluate cell proliferation, death and density, as well as cell adhesion, elongation, myoblast fusion and the formation of multinucleated myotubes.

Initially, primary cultures of chick myogenic cells are composed by two major mononucleated cell types, myoblasts, and fibroblasts. Under phase contrast microscopy, mononucleated myoblasts are bipolar, with a central cytoplasmic expansion containing a phase dark nucleus, and two cytoplasmic processes. Differently, mononucleated fibroblasts are triangular or pleomorphic, with a less dense nucleus and a granular cytoplasm (Fear, 1977). In addition, chick myoblasts and fibroblasts can be also distinguished by their nuclear morphology and DNA content as revealed by a fluorescent nuclear probe, such as DAPI. In DAPI-stained chick myogenic cultures, muscle fibroblasts show large flattened pale nuclei, whereas myoblasts exhibit small round bright nuclei (Yamashita et al., 2017). The use of specific protein markers can also aid to identify each cell type. Myoblasts are positive for the intermediate filament desmin (Costa et al., 2004), whereas fibroblasts are negative for desmin and positive for the vesicle-associated protein flotillin-2 (Possidonio et al., 2014b). Thus, the percentage of myoblasts can be calculated by the double-staining of 24-h cultures with a nuclear dye, such as DAPI, and a muscle-specific protein marker, such as an anti-desmin antibody. Subsequently, it is only necessary to count the number of desmin-positive mononucleated cells out of the total number of cells in the field. On average, myoblasts make up 80% of each culture and non-myogenic cells comprise 20%. Additional immunolabeling with an antibody against flotillin-2 can confirm that all non-myogenic cells in these cultures are fibroblasts (Possidonio et al., 2014b). If necessary, it is possible to treat 24-h chick muscle cultures with anti-mitogenic drugs such as 1 μg/mL of arabinoside cytosine (ara-c) to reduce the number of fibroblasts. As an alternative to further decrease the number of fibroblasts in these cultures, is to use the differential adhesion characteristics of myoblasts and fibroblasts to the culture dish surface. More specifically, myoblasts are more loosely attached to the plastic surface than fibroblasts (Kaighn et al., 1966; Richler and Yaffe, 1970). Nevertheless, fibroblasts are important to muscle cell differentiation in culture, since they secrete many components of the extracellular matrix, such as collagen, laminin, and fibronectin, as well as numerous growth factors (Chapman et al., 2016). Their use in chick muscle cultures mimic the *in vivo* environment, where bundles of muscle fibers are in close association with fibroblast enriched-connective tissue (in the endomysium, perimysium, and epimysium). Chick muscle cells can grow up to 15 days in culture and after that, myotubes begin to detach from the culture dish because of their size and intense contraction.

Chick myogenic cells are platted on rat tail collagen-coated dishes. Collagen I is the main component of collagen fibers found in rat tails and it is also one of the main components found in the connective tissue layers (endomysium, perimysium, and epimysium) that envelops muscle fibers. It has been shown that collagen provides an ideal extracellular matrix substratum for the adhesion, survival, growth, and differentiation of chick muscle cells grown *in vitro* (Haba et al., 1975). Collagen I enriched solutions can be easily prepared from rat tails (either frozen or freshly isolated from rats). Briefly, rat tails (between 3 and 10) are soaked in a Balanced Salt Solution (BSS, Gibco, United States) for 24 h at 4°C. Then, they are placed in 95% ethanol solution and collagen fibers are isolated by pulling them out of the tails with the use of scissors and forceps. Finally, the isolated collagen fibers are trimmed into small pieces and immersed in 1% acetic acid solution (60–100 mL per rat tail) for 48 h under at 4°C, for the degradation of collagen fibers. This solution is then centrifuged at 5,000 rpm for 15 min and the supernatant is collected and stored in 50 mL tubes at −20°C for up to 2 years. The viscosity of the final collagen solution needs to be verified and further dilution with acetic acid are usually needed prior to use in cell dishes.

## A Bibliometric Glimpse of the Contributions of Chick Cell Culture to the Study of Skeletal Myogenesis

**FIGURE 5:**
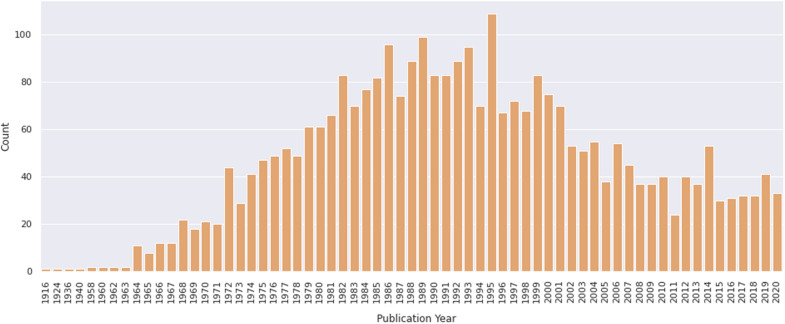
Number of papers on primary culture of embryonic chick myogenic cells per year. Distribution of publications retrieved from PubMed. The retrieved dataset only included papers until the year of 2020.

To evaluate the contribution of chicken cell culture to the field of skeletal myogenesis, we performed exploratory analyses of data retrieved from the PubMed^1^ database. The query was performed on January 31, 2021 by using the following descriptors: (development OR differentiation OR myogenesis) AND (muscle OR myogenic OR muscular) AND (chick OR chicken) AND (vitro OR culture). We found a total of 3032 articles in a period that spanned the years 1916–2020 (Figure 5). The early years (1916–1963) exhibited only one or two articles published per year, whereas years 1964–1995 showed a continuous and significant increase in the number of publications, which reached a peak in 1995 with more than 100 papers in a single year (Figure 5). In turn, the years 1996–2011 revealed a decline in publications per year, until reaching a fairly regular plateau of 30–40 papers per year in between 2012 and 2020. The higher number of publications observed from 1972 to 1989 may be associated with the extensive use of chick cell culture in seminal studies of muscle development. Despite a relative drop in numbers of papers in recent years, it is relevant to note that the culture of chick muscle cells continues to be used nowadays. This trend is probably related to the tweaking of new molecular and cellular biology techniques to be used in chick cell cultures. Among them, we can cite tools to study gene function through cell transfection, such as plasmids for reporter-based assays (such as TOP-Flash assay for the Wnt/beta-catenin pathway), gene overexpression, siRNA-dependent knockdown, or CRISPR-based gene editing (Mermelstein et al., 2007; Possidonio et al., 2016; Yamashita et al., 2017; Bagri et al., 2020; de Andrade Rosa et al., 2020).

**FIGURE 6:**
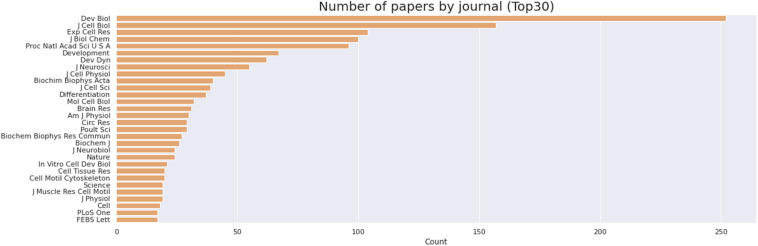
Journals that published most papers on primary culture of embryonic chick myogenic cells. Number of papers by journal, as indicated. The retrieved PubMed dataset only included papers until the year of 2020.

**FIGURE 7:**
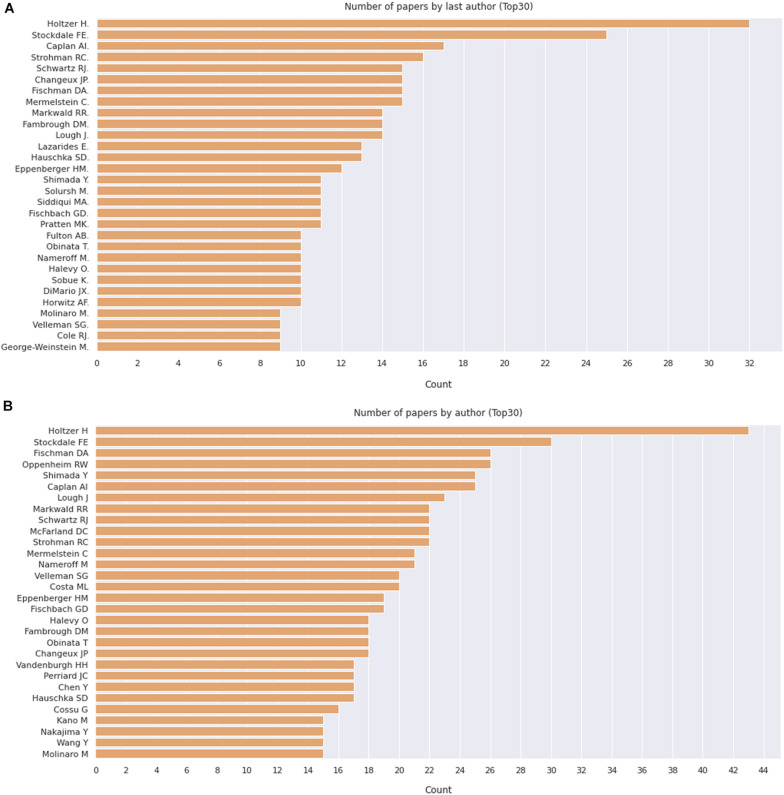
Number of apers by last author. **(A)** Number of papers by journal, as indicated. **(B)** Pie chart depicting the relative proportion of journals grouped by the number of published papers on the subject. The retrieved PubMed dataset only included papers until the year of 2020.

**FIGURE 8:**
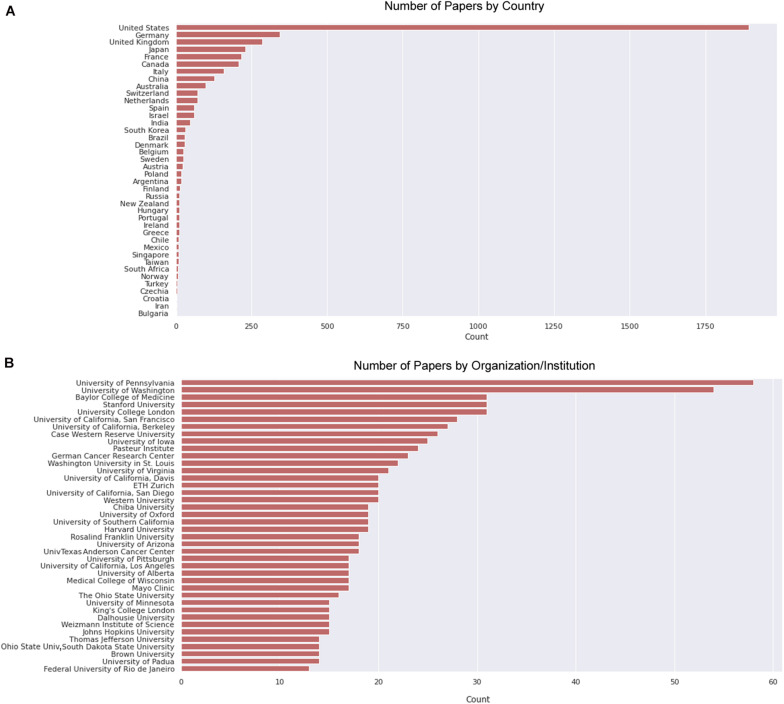
Countries and institutions that harbored most papers on primary culture of embryonic chick myogenic cells. **(A)** Number of papers by country of the leading institution, as indicated. **(B)** Number of papers by leading institution. We defined the leading institution as the first affiliation depicted in the retrieved Dimensions dataset, which included publications until the year of 2020.

We then delve into the main players of the chick muscle cell field. The scientific journals that published most of the studies using culture of chick muscle cells in the evaluated period were Developmental Biology, Journal of Cell Biology, Experimental Cell Research, Journal of Biological Chemistry, Proceedings of the National Academy of Sciences (PNAS), Development, and Developmental Dynamics. The top 30 journals that published papers on *in vitro* chick myogenesis are on the fields of developmental biology, cell biology, and biochemistry, in addition to a few multidisciplinary journals (Figure 6). However, more than half of the journals that communicated papers which used chick cell culture published only one paper, whereas about 9% of the identified journals were responsible for reporting more than 10 papers in the period (Figure 6). In addition, we examined the researchers involved in these publications. The largest contributor in number of articles using culture of chick muscle cells was Professor Howard Holtzer, from the University of Pennsylvania (United States), with 43 publications (of these, 32 as the last author; Figure 7). Then, Professor Frank E. Stockdale, from Stanford University (United States) also used this model in 30 studies (of these 25, as the last author). A list of the top 30 researchers that used culture of chick cells and their respective numbers can be found in Figure 7. The list of the top 30 researchers is in accordance with the lists of organizations/institutions and countries that have harbored the largest number of studies using chick muscle cells (Figure 8). Many of the researchers found in the list of major contributors of the chick muscle research field were members of the Pennsylvania Muscle Institute^2^ (PMI, United States), which is an interdisciplinary group of research investigators whose goal is to discover the mechanisms of muscle function, muscle disease, muscle contraction and development.

**FIGURE 9:**
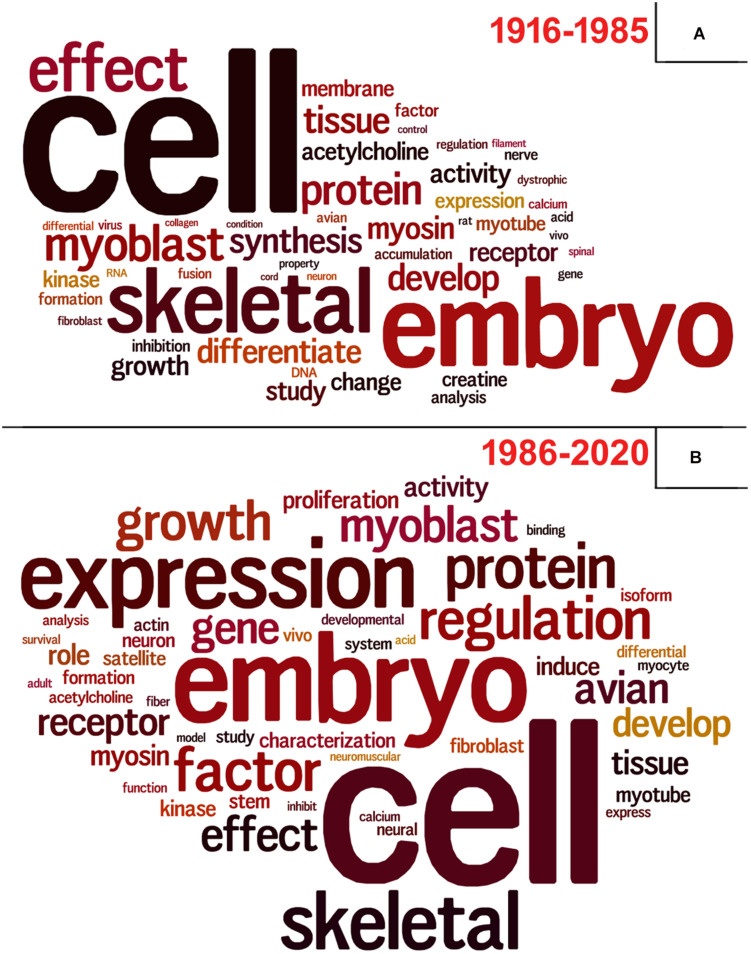
Vocabulary analysis of the articles using chick muscle cultures. **(A)** A word cloud was generated using the titles of articles on primary culture of embryonic chick myogenic cells. This method gives greater prominence to frequent words. **(B)** Word frequency list. The retrieved PubMed dataset only included papers until the year of 2020.

To perform these analyzes, we used data retrieved from the Dimensions database^3^ using the same descriptors as query, since PubMed did not provide this information. Lastly, we questioned ourselves what subjects/themes were most used in articles using chick muscle cell cultures. To answer this question, we gathered all the titles from the retrieved papers and examined the word frequency upon lemmatization (Peng et al., 2020; Yuhao et al., 2020). We separated our analysis in two groups: articles published between 1916–1985 and 1986–2020, to be able to compare possible word differences over the years. As expected, the used descriptors (“cell,” “embryo,” and “skeletal”) were found among the most common words (Figure 9). Should we disregard them for subsequent analysis, we observed some interesting differences over the years in the use of specific words in papers in the chick muscle cell culture field. A higher frequency of cellular process-related words (e.g., “expression,” “regulation,” “growth,” “gene,” and “proliferation”) was observed in the later years (1916–2020), whereas a higher frequency of words related to molecules, structures or organelles (“acetylcholine,” “membrane,” “creatine,” “DNA,” and “RNA”) was observed in the early years (1986–1985) (Figure 9). These data points to the evolution of the muscle biology field from mostly descriptive studies toward a mechanistic understanding of muscle cell structure and function, including how different intracellular signaling pathways and networks regulate gene expression and cell physiology in muscle.

## Summary

The collection of data presented here point to the robustness of chick myoblast culture as a tool for the understanding of the role of different molecules and signaling pathways during the skeletal muscle differentiation program. We expect that by describing the major findings, and their advantages, of the studies using the embryonic chick myogenic model we will foster new studies on the molecular and cellular processes involved in muscle proliferation and differentiation that are more similar to the actual *in vivo* condition than the muscle cell lines.

## Author Contributions

CM wrote the manuscript. AJ and MC revised the manuscript critically. AJ, CM, and MC performed the bibliometric analysis and prepared the figures. All authors contributed to the article and approved the submitted version.

## Conflict of Interest

The authors declare that the research was conducted in the absence of any commercial or financial relationships that could be construed as a potential conflict of interest.

## Footnotes

1 https://pubmed.ncbi.nlm.nih.gov/

2 https://www.med.upenn.edu/pmi/

3 https://app.dimensions.ai/discover/publication

## Supplementary Material

Supplementary Figure 1 Myoblast fusion dynamics. Movie showing several myoblasts in a row during fusion in a 24-h chick myogenic culture. Note that fusion tended to follow end-to-end rather than side-to-side alignment of myoblasts.
